# Suicidal Behavior in Adolescents: A Latent Class Analysis

**DOI:** 10.3390/ijerph17082820

**Published:** 2020-04-19

**Authors:** Adriana Díez-Gómez, Alicia Pérez-Albéniz, Carla Sebastián-Enesco, Eduardo Fonseca-Pedrero

**Affiliations:** 1 Department of Educational Sciences, University of La Rioja, 26004 Logroño, Spain; adriana.diez@unirioja.es (A.D.-G.); eduardo.fonseca@unirioja.es (E.F.-P.); 2 Programa Riojano de Investigación en Salud Mental (PRISMA), 26004 Logroño, Spain; carla.sebastian@gmail.com; 3 Department of Research and Psychology in Education, University Complutense of Madrid, 28223 Madrid, Spain; 4 Department of Psychiatry, Centro de Investigación Biomédica en Red de Salud Mental (CIBERSAM), 33006 Oviedo, Spain

**Keywords:** suicide, suicidal behavior, adolescents, risk, latent classes, typologies

## Abstract

The main goal of the present study was to identify and validate latent classes of suicidal behavior in a representative sample of adolescents. The sample comprised a total of 1506 students, including 667 males (44.3%), selected through a sample stratified by clusters. The mean age was 16.15 years (SD = 1.36). The instruments used evaluated suicidal behavior, positive and negative affect, emotional and behavioral problems, prosocial behavior, and subjective well-being. Using the Paykel Suicide Scale, the latent class analysis identified four homogeneous subgroups: “low risk”, “suicidal act”, “suicidal ideation”, and “high risk for suicide”. These subgroups presented a differential pattern in terms of their social-emotional adjustment. The subgroups with the highest theoretical risk showed lower scores on subjective well-being and positive affect as well as higher scores on emotional and behavioral problems and negative affect compared to the non-risk subgroups. This study contributes to an understanding of the typologies of suicidal behavior among adolescents and the relationship with psychopathological adjustment. Ultimately, these findings may promote the development or improvement of early detection and prevention strategies in the suicidal behavior field in order to reduce the socio-economic burdens associated with suicide in young populations.

## 1. Introduction

Suicidal behavior is defined as a fatal self-harming act with the intention to die [1]. It is a heterogeneous, multicausal, and multidimensional construct [2]. Suicidal behavior encompasses different manifestations such as suicidal ideation (ideas and/or thoughts of death), suicidal communication (both verbal and non-verbal formulation), threats, suicidal gesture and/or self-injury acts and the suicidal act itself [3,4,5,6].

The different types of suicidal behavior are common among young people aged 15 to 29 years [7]. Indeed, suicide is the second leading cause of death in young populations [8]. Suicidal behavior among children and adolescents is, however, different to that reported in adult populations [9]. Prevalence rates seem to vary not only by age but also by gender. For instance, the prevalence of active suicidal ideation among adolescents ranged from 20% to 30% [10,11,12]. In a meta-analysis, Lim et al. [13] found that the life prevalence and the 12 month prevalence of suicide attempts in adolescents was 6% (95% CI: 4.7%–7.7%) and 4.5% (95% CI: 3.4%–5.9%), respectively. Regarding suicide ideation, life prevalence corresponds to 18% of adolescents (95% CI: 14.2%–22.7%) and 12 month prevalence to 14.2% (95% CI: 11.6%–17.3%). With respect to gender, studies show that in samples of adolescents and young adults, females present a higher risk of suicide attempt (OR 1.96; IC 95% 1.54–2.50), whereas males present a higher risk of suicide consummation (HR 2.50; IC 95% 1.8–3.6) [14]. In Spain, the prevalence of suicidal ideation in the adolescent population is approximately 30%, while the prevalence of suicide attempts is approximately 4% [12,15].

Several risk factors have been associated with suicidal behavior [16,17]. Depressive symptoms are among the most commonly reported factors in suicidal behavior [18]. However, anxiety symptoms [19,20], affective disorders [19,20], disruptive behavior [18], and substance disorders [18] were found to be influential risk factors when analyzing adolescent suicide. In addition, young people with suicidal behavior (e.g., ideation, previous suicide attempts) report, among others, greater behavioral problems, substance use, and risk behaviors, as well as a lower quality of life, [1,12].

One of the current research lines focuses on identifying and classifying individuals as a function of their suicide risk. The reliable identification and detection of high-risk groups of individuals as well as the analysis of their behavioral, cognitive, and emotional characteristics may contribute to the understanding and prevention of suicide. New psychometric approaches, such as latent class analysis (LCA), enable the determination of how individuals are grouped together according to a particular set of symptoms, behaviors, or traits [21,22]. This psychometric approach has potential benefits in detecting the risk of suicidal behavior. From this approach, one might obtain, among other important things, data-driven identification, a broader understanding of the behavioral patterns underlying each suicide-risk subgroup, and eventually, the eradication of the widespread assumption that suicidal behavior corresponds to a single type of behavior. 

The latent class (LC) approach was used in previous studies on suicidal behavior for different purposes. Some works focused on identifying the precipitating thoughts of death and suicide among adolescents [23,24], or recognizing functioning patterns of risk factors [25,26], whilst others aimed at predicting suicidal behaviors [27], or exploring the subtypes of psychiatric disorders that were strongly associated with suicidal thoughts or behaviors [28,29]. At present, there are several studies that examined the heterogeneity among individuals with varying histories of suicidal behavior [9,24,30,31,32,33]. Mixture modelling has been employed in adolescent populations [32,33,34,35,36]. For instance, Jiang et al. [32] identified four groupings for a representative sample of adolescents: (1) emotionally healthy, (2) considered and planned suicide, (3) attempted suicide and (4) planned and attempted suicide. Crucially, the authors found that adolescents with the highest risk of suicide also presented higher levels of negative affect. 

Findings on the classification of individuals according to suicide risk are heterogeneous, and strongly depend on the theoretical delimitation of suicidal behavior [3,4,5,6], demographic variables (e.g., age, gender, nationality), the empirical design (longitudinal, transversal), and the measuring instrument used. To the best of our knowledge, no previous studies have analyzed the latent classes of suicidal behavior using the Paykel Suicide Scale (PSS) [37] and provided validated data in relation to multiple indicators of social-emotional adjustment during adolescence. Within this research framework, the main goal of the present study was to identify subgroups of suicidal behavior in a large sample of adolescents of the general population through LCA. In addition, the second goal was to validate the latent LC model by analyzing whether the subgroups identified have a differential pattern in terms of their emotional and behavioral problems, subjective well-being, prosocial behavior, and positive and negative affect. 

## 2. Method

### 2.1. Participants

Stratified random cluster sampling was conducted at the classroom level in an approximate population of 15,000 students selected from a region located in northern Spain. The students were from various public and state-subsidized secondary schools and vocational training centers, as well as from a range of socio-economic levels. The strata were created on the basis of geographical zone (East, West, and Centre) and educational stage (compulsory–to age 16–and post-compulsory), where the likelihood of inclusion depended on the number of students in the school. 

Participants were 1506 students, including 667 males (44.3%), from 34 educational centers and 98 classrooms. The mean age was 16.5 years (*SD* = 1.36), with an age range from age 14 to 19. The distribution by age was: 14-year-olds (*n* = 200; 13.3%), 15-year-olds (*n* = 313; 20.8%), 16-year-olds (*n* = 381; 25.3%), 17-year-olds (*n* = 365; 24.2%), 18-year-olds (*n* = 174; 11.6%), and 19-year-olds (*n* = 73; 4.8%). 

The nationality distribution of the participants was as follows: 89.9% Spanish, 3.7% Latin American (Bolivia, Argentina, Colombia, and Ecuador), 0.7% Portuguese, 2.4% Romanian, 1% Moroccan, 0.7% Pakistani, and 2% from other nationalities.

### 2.2. Instruments

The instruments used in the study assess variables that, according to previous works, are closely related to suicidal behavior [12,38]. 

*The Paykel Suicide Scale* (PSS) [37]. The PSS is a self-report tool designed for the evaluation of suicidal behavior. The tool consists of a total of 5 items (1. Have you ever felt that life is not worth the trouble? 2. Have you ever wished you were dead? For example, going to sleep and not wanting to get up. 3. Have you ever thought of taking your life, even though you were really not going to do so? 4. Have you reached the point when you really considered taking your life or made plans about how you would do so? 5. Have you ever tried to take your own life?), with a dichotomous response system, i.e., Yes/No questions (score as 1 and 0, respectively). The scores range from 0 to 5. The time frame to which the questions refer is the last year. Higher scores are related with high severity on suicidal ideation. The Spanish adaptation of the PSS has demonstrated adequate psychometric properties [12,15].

*The Strengths and Difficulties Questionnaire* (SDQ) [39]. The SDQ is a self-report questionnaire that is widely used for the assessment of different emotional and behavioral difficulties related to mental health in adolescents. The SDQ consists of a total of 25 statements distributed across five subscales: emotional symptoms, conduct problems, hyperactivity, peer problems, and prosocial behavior. The first four subscales yield a total difficulties score. In this study, we used a Likert-type response format with three options: 0 (*not true*), 1 (*somewhat true*) and 2 (*certainly true*). The validated Spanish version of the SDQ was used in the present study [40].

*The Personal Well-being Index—School Children* (PWI-SC) [41]. The index comprises eight items, with response options ranging from 0 (*completely dissatisfied*) to 10 (*completely satisfied*). The PWI-SC items assess subjective satisfaction within a specific area of life in a relatively generic and abstract way. The first item on the scale analyzes “life as a whole”. The other seven items assess satisfaction with different life domains: standard of living, health, life achievements, relationships, safety, community connectedness, and future security. The validated Spanish version of the PWI-SC was used in the present study [38]. 

*The 10-item Positive and Negative Affect Schedule for Children* (PANAS-C) [42]. This questionnaire consists of 10 items and two factors designed to measure positive affect (PA) and negative affect (NA), respectively. Five items assess PA through the following adjectives (happy, lively, happy, energetic, and proud) and the other five items assess NA (depressed, angry, fearful, scared, and sad). Children/adolescents have to indicate the extent to which they have experienced each emotion in the past few weeks on a 5-point Likert scale ranging from 1 (*very slightly or not at all*) to 5 (*extremely or very much*). The validated Spanish version of PANAS-C was used in the present study [38].

*The Penn Matrix Reasoning Test* (PMRT) [43,44]. This is a task in the Penn Computerized Neurocognitive Battery-Child version developed to measure non-verbal reasoning (using matrix reasoning problems as used in the Raven’s Progressive Matrices Test) within the complex cognitive domain. This task, which is composed of 20 items, may be considered as an estimated Intelligence Quotient (IQ). The battery includes different neurobehavioral indicators with different tasks adapted to guarantee psychometric properties and its linkage to brain systems for children [43,44]. The validated Spanish version of this neurocognitive battery was used in previous studies [45].

*The Family Affluence Scale-II* (FAS-II) [46]. Socio-economic status was measured using a 4-item child-appropriate measure of family wealth, with scores ranging from 0 to 9. Previous international studies have demonstrated its adequate psychometric properties [46]. The validated Spanish version of the FAS-II was used in previous studies [38].

*The Oviedo Infrequency Scale* (INF-OV) [47]. The INF-OV scale was administered to the participants to detect those who responded in a random, pseudorandom or dishonest manner. The INF-OV instrument is a self-report composed of 12 items in a 5-point Likert scale format ranging from 1 (*completely disagree*) to 5 (*completely agree*). Students with more than three incorrect responses on the INF-OV scale were eliminated from the sample. The validated Spanish version of the INF-OV scale was used in this study [47].

### 2.3. Procedure

The research was approved by the Educational Government of La Rioja and the Ethical Committee of Clinical Research of La Rioja (CEICLAR). The tests and neurocognitive battery were administered collectively, through personal computers, in groups of 10 to 30 students, during normal school hours and in a classroom specially prepared for this purpose. Administration took place under the supervision of the researchers trained in a standard protocol. No incentive was provided for their participation. For participants under 18, parents were asked to provide a written informed consent in order for their child to participate in the study. Participants were informed of the confidentiality of their responses and of the voluntary nature of the study. 

### 2.4. Data Analysis

First, we calculated descriptive statistics for all measures. Second, in order to test for the existence of discrete groups (classes) with similar psychometric profiles, we conducted LCA using the dichotomous items of the PSS.

In LCA, models are compared to determine the optimal number of classes (i.e., class enumeration), beginning with evaluating the fit of a one-class model and incrementally adding latent classes until the best class solution has been satisfied. Model selection is based on consideration of several fit indices including information criteria and likelihood ratios. For the information criteria, we used the Akaike information criterion (AIC) [48], the Bayesian information criterion (BIC) [49], and the sample size-adjusted BIC (ssaBIC) [50]. With regard to the information criterion statistics, lower values indicate a better fit. We considered the Lo–Mendell–Rubin adjusted likelihood ratio test (LRT) [26]. The likelihood ratios of the *k*-1 and *k* class models test the null hypothesis to determine whether there is a statistically significant difference. Thus, a *p* < 0.05 suggests that the *k* class model is a better fitting model than the *k*-1 class model, whereas a *p* > 0.05 suggests that *k*-1 class solution is preferred in terms of accurately reflecting the data. We can further assess whether we have chosen the right number of classes using the bootstrapped parametric likelihood ratio test. A standardized measure of entropy was also computed. The entropy measure (values ranging from 0 to 1) assesses the relative accuracy of participant classifications, with higher values indicating better separation of the identified groups [51].

Fourth, the association of latent class membership with the SDQ, PANAS-C, and PWI-SC scores was analyzed using multivariate analysis of covariance (MANCOVA). Gender, estimated IQ, and socio-economic status were used as covariates. Partial eta squared (η^2^) was used as index of effect size. 

SPSS 22.0 (IBM, New York, NY, USA) [52] and Mplus 7.4 (Muthén & Muthén, Los Angeles, CA, USA) [53] were used for these analyses.

## 3. Results

### 3.1. Identification and Delimitation of the Latent Classes of Suicidal Behavior

The total percentages for the PSS scores were as follows: 0 = 58.6%, 1 = 16.5%, 2 = 9.8%, 3 = 8.2%; 4 = 4.8%, and 5 = 2.0%.

Table 1 displays the fit indices resulting from the different estimated LC models. As noted, the four-class model was the best fitting solution compared to the one-, two-, three- and five-class models. Comparatively, the four-class model presented the highest entropy value, a statistically significant LMR-A *p*-value, and the lowest AIC and BIC values. The three-class model also showed adequate goodness-of-fit indices, although the entropy value was lower, and the AIC and BIC indices were slightly higher compared to the four-class model.

Membership for the four-class solution was as follows: LC1 *n* = 1130 (75.03%), LC2 *n* =15 (1.00%), LC3 *n* =104 (6.9%), and y LC4 *n* = 73 (17.06%). The mean probability of membership for each LC was 0.96, 0.99, 0.99 and 0.94, respectively. This result reveals the existence of a clear differentiation among the four subgroups of participants—that is, a participant grouped into a particular LC has a high probability of belonging to that class, and no other. 

**Figure 1:**
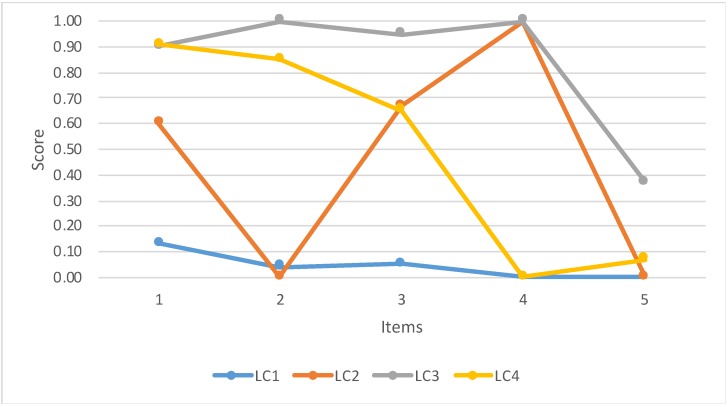
Results of the four latent class (LC) model.

Figure 1 shows the scores on the PSS items for the resulting profiles of individuals. Latent class 1 (LC 1) consisted primarily of individuals with low scores on the PSS items and, thus, was called “low risk”. Latent class 2 (LC 2) includes individuals with high scores on item 3 and 4 and was labelled as “suicidal act”. Latent class 3 (LC 3) includes participants with high scores on all PSS items and was, thus, called “high suicide risk”. Lastly, latent class 4 (LC 4) consisted of individuals with high scores on item 1, 2, and 3 and was labelled as “suicidal ideation”. Comparatively, latent class (LC 3) presented a higher mean PSS total score (*p* < 0.01). 

### 3.2. Validation of the Latent Classes of Suicidal Behavior: Social-Emotional Adjustment

**Figure 2:**
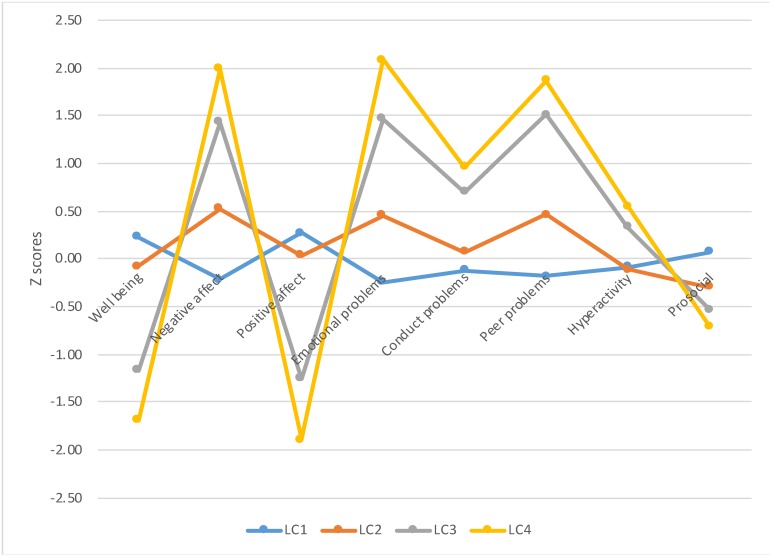
Social-emotional adjustment across latent classes.

A MANCOVA revealed statistically significant differences when considering all variables on class membership as fixed factors (*Wilk’s λ* = 0.666, *F*_(24,4327)_ = 27,112; *p* < 0.001, partial *η*^2^ = 0.127). Table 2 shows the mean scores, standard deviations, *p*-values, and effect sizes resulting from the analyses. Figure 2 displays the distribution of z-scores derived from the mean scores. In some cases, the estimated effect sizes were high. A clear differentiation was found among groups of participants, which suggests that there are distinct behavioral patterns of social-emotional adjustment underlying the latent classes.

The “high suicide risk” group presented higher scores on emotional and behavioral problems and lower scores on prosocial behavior compared to the rest of the groups. With regard to subjective well-being, LC 3 and LC 4 displayed lower levels than did the remaining classes. For the negative and positive affect, differences among latent classes were clear cut, with LC 3 presenting the lowest level of positive affect and one of the highest mean scores on negative affect (*p* < 0.01). 

## 4. Discussion

Suicide is a social-health problem among young people worldwide. Characterization of protective and high-risk subgroups might propel a deeper understanding of suicidal behavior subtypes. Furthermore, identifying suicide-risk subgroups can also help to implement programs aimed at promoting emotional well-being and to develop specific target interventions. Thus, the aim of this study was to identify homogeneous empirically derived subgroups of individuals with suicide risk in a representative sample of adolescents. Additionally, we explored whether participants grouped into the LC presented a distinct pattern in terms of their emotional and behavioral problems, subjective well-being, prosocial behavior, and positive and negative affect. 

First, using the Paykel Suicide Scale (PSS), the LC analysis identified four distinct groups: “low risk—healthy”, “suicidal act”, “suicidal ideation”, and “high suicide risk”. These findings closely match those of previous studies using the LC approach in both adolescent and adult populations [23,24,25,27,28,29,54]. Previous studies show an empirically derived classification that partially coincides with the present study [32,35,36,55]. For instance, King et al. [35] identified five profiles of adolescents based on their past history of suicide attempts, suicide ideations endorsed in the past month, depression, alcohol and drug use, aggressive impulses, and past history of physical and/or sexual abuse. Although the number of groups were different to that of the present work, the authors found four groups of individuals on the basis of the suicide attempts (acts), suicide ideation, and the risk of suicide when considering the association between suicidal behavior and other related variables. In another study, Xiao et al. [33] acknowledged four distinct groups of adolescents according to 13 health indicators and observed that those groups with lower levels of engagement in health-promoting behaviors also displayed higher risks of suicide. Likewise, research on adults at suicide risk using a LC approach also showed distinct subgroups of individuals [31,56,57]. In particular, the study by Ma and collaborators [56] identified four different groups of adults who endorsed suicide ideation in the past on the basis of individuals’ age and the severity of their mental health symptoms: (1) low levels of thwarted belongingness and low capability of suicide; (2) low levels of thwarted belongingness and high capability of suicide; (3) high levels of thwarted belongingness and low capability of suicide; (4) high levels of thwarted belongingness and high capability of suicide.

Second, the empirically derived groups of suicidal behavior exhibited distinct underlying patterns of psychological and social-emotional adjustment. Specifically, the group with high theoretical suicide risk reported higher levels of behavioral and emotional problems along with lower scores on prosocial behavior, emotional welfare, and positive affect. To the best of our knowledge, no previous studies have analyzed the risk of suicide among adolescents with a LC approach and a possible relationship with social-emotional adjustment. Thus, it is difficult to make comparisons with other studies. However, several studies have provided a taxonomy of adolescents with suicidal ideation based on mental health problems. For example, Jung et al. [55] developed a study in which three latent profiles were identified as a function of internalizing and externalizing problems. Similarly, studies using an LCA approach showed a relationship between the risk of suicide and other variables related to lifestyle, such as pattern of sleep, physical exercise, and drug use [2,32,33,34,35]. The results revealed that health-promoting behaviors, a regular diet, and moderate exercise had a protective impact on adolescents’ suicidal behaviors by decreasing the ultimate suicide risk [33]. In the same line, the work by King et al. [35] showed that adolescents with a higher risk of suicide reported higher levels of alcohol and drug consumption and aggressive impulses.

Crucially, results of studies like the present one may enable effective interventions directed at adolescents presenting high scores on suicidal ideation and on negative affect symptomatology (class 4) before they transition to individuals with a serious risk of suicidal attempt (LC 3). Different types of cognitive interventions aimed at reducing suicidal thoughts and ideas can be conducted in the latent classes [58], and this can ultimately prevent suicidal behavior from progressing toward attempted suicide, as successfully shown by previous studies [59,60]. Indeed, prior research has found that a death wish and suicidal ideation are important predictor variables of suicide [61]. 

We also observed a group of individuals with high scores on suicidal acts (item 3 and 4 of the PSS), but not on suicidal ideation. Several studies provided convergent evidence in this respect, evincing a distinct group formed by adolescents with high rates on attempted suicide, but moderate scores on thoughts of death or suicidal ideation [62]. In our particular study, we could identify a subgroup within LC2 with NSSI. The so-called “non-suicidal self-injurious” behaviors (NSSI) characterized by self-injurious acts without a suicidal ideation are not pathological by themselves but have a risk impact on individuals’ suicide disposition. These types of behaviors might act as a self-regulatory mechanism for adaptation to the environment [62]. Potential causes underlying the NSSI behaviors are diverse, ranging from relieving intense distressing affects by through physical pain, gaining attention so that other people can see their distress, manipulating others’ behavior through threats, and fitting in socially with peers who self-injure [63]. Identifying young individuals with NSSI behaviors may enable the development of more efficient interventions aimed at promoting emotional competence and coping strategies. Ultimately, this can enable individuals to face distress adaptively and prevent self-injury from becoming the only solution to reduce distressing affect [64]. 

The results of the present study should be interpreted in light of the following limitations. First, although the sample selected was representative, all participants came from a single Spanish region, La Rioja. For generalization purposes, future research should include individuals from different Spanish regions. Second, the findings were based on a self-report by adolescents. As is well known, self-report instruments present problems in terms of social desirability and response bias, which might be especially important in these age groups. Thus, further studies should also incorporate instruments from other reports in order to ensure the validity of the findings as well as directly measure the NSSI. Third, future research needs to provide data on similar populations using the same instrument to confirm that the current results are replicable. Finally, given that this is a cross-sectional study, no causal inference can be drawn. Despite such limitations, the present work contributes to the analysis and understanding of suicidal behavior in adolescence. In contrast to previous studies, we used an innovative psychometric method, LCA, which generates meaningful grouping of adolescents at heightened risk for suicide. In addition, a representative and random sample of the adolescent general population was used. Furthermore, multiple social-emotional indicators to validate the LC approach was used. 

Beyond the latter considerations on prevalence studies, future research should focus on designing suicide intervention programs that specifically target the strategies demanded by each risk group of adolescents. New psychometric approaches such as network analyses may contribute to a broader understanding of the etiological mechanisms underlying suicidal behavior. In addition, new ways to measure suicidal behavior in the real world (e.g., ambulatory assessment) [65] as well as the development of new evidence-based suicide prevention programs are relevant lines of future research [66]. 

## Figures and Tables

**Table 1:** Fit indices for the latent class models on suicidal behavior for the total sample.

Models	Log-Likelihood	AIC	BIC	ssaBIC	Entropy	LMR-A	LMR-A *p*
1 class	−3238.22	6486.44	6513.03	6497.15	-		
2 classes	−2558.36	5138.73	5197.22	5162.27	0.87	1329.44	0.001
3 classes	−2508.76	5051.52	5141.92	5087.91	0.87	96.994	0.001
4 classes	−2500.41	5046.81	5169.11	5096.04	0.91	16.338	0.001
5 classes	−2500.13	5058.26	5212.46	5120.33	0.81	0.541	0.189

Note. AIC = Akaike information criterion; BIC = Bayesian information criterion; ssaBIC = sample size-adjusted BIC; LMR-A = the Lo–Mendell–Rubin adjusted likelihood ratio test.

**Table 2:** Mean scores on social-emotional adjustment as a function of the resulting latent classes.

Scores	LC1 Low Risk		LC2 Suicidal Act		LC3 High Suicide Risk		LC4 Suicidal Ideation		*F*	*p*	Partial *η*^2^
	Mean	SD	Mean	SD	Mean	SD	Mean	SD			
PWI-SC	0.22	0.84	0.31	1.09	−1.08	1.2	−0.53	1.07	98.217	<0.001	0.164
Negative affect	−0.22	0.91	0.74	0.87	0.91	0.92	0.56	0.98	83.269	<0.001	0.143
Positive affect	0.27	0.71	0.24	1.08	−1.28	1.35	−0.64	1.21	152.074	<0.001	0.233
SDQ Emotional symptoms	−0.24	0.88	0.69	0.92	1.02	0.96	0.61	0.96	105.874	<0.001	0.175
SDQ Conduct problems	−0.12	0.95	0.19	0.74	0.63	1.2	0.26	1	28.457	<0.001	0.054
SDQ Peer problems	−0.19	0.85	0.65	1.3	1.05	1.3	0.36	1.06	74.059	<0.001	0.129
SDQ Hyperactivity	−0.09	1	−0.02	1.17	0.44	0.92	0.22	0.96	13.367	<0.001	0.026
SDQ Prosocial behavior	0.07	0.94	−0.36	1.08	−0.24	1.24	−0.17	1.09	9.078	<0.001	0.018

Note. LC = Latent Class; SDQ = the Strengths and Difficulties Questionnaire; PWI-SC = the Personal Well-being Index—School Children.

## References

[1] Turecki G., Brent D.A. (2016). Suicide and suicidal behaviour. Lancet.

[2] Bernanke J., Galfalvy H., Mortali M.G., Hoffman L.A., Moutier C., Nemeroff C.B., Stanley B.H., Clayton P., Harkavy-Friedman J., Oquendo M.A. (2016). Suicidal ideation and behavior in institutions of higher learning: A latent class analysis. J. Psychiatr. Res..

[3] Anseán A. (2014). Suicidios: Manual de Prevención, Intervención y Postvención de la Conducta Suicida.

[4] Goodfellow B., Kõlves K., de Leo D. (2018). Contemporary Nomenclatures of Suicidal Behaviors: A Systematic Literature Review. Suicide Life-Threat. Behav..

[5] O’Connor R.C., Platt S., Gordon J. (2011). International Handbook of Suicide Prevention: Research, Policy and Practice.

[6] O’Connor R.C., Pirkis J. (2016). The International Handbook of Suicide Prevention.

[7] Lunde I., Reigstad M.M., Moe K.F., Grimholt T.K. (2018). Systematic literature review of attempted suicide and offspring. Int. J. Environ. Res. Public Health.

[8] Gore F.M., Bloem P.J.N., Patton G.C., Ferguson J., Joseph V., Coffey C., Sawyer S.M., Mathers C.D. (2011). Global burden of disease in young people aged 10–24 years: A systematic analysis. Lancet.

[9] Goldston D.B., Erkanli A., Daniel S.S., Heilbron N., Weller B.E., Doyle O. (2016). Developmental trajectories of suicidal thoughts and behaviors from adolescence through adulthood. J. Am. Acad. Child Adolesc. Psychiatry.

[10] Evans E., Hawton K., Rodham K. (2004). Factors associated with suicidal phenomena in adolescents: A systematic review of population-based studies. Clin. Psychol. Rev..

[11] Kokkevi A., Rotsika V., Arapaki A., Richardson C. (2012). Adolescents’ self-reported suicide attempts, self-harm thoughts and their correlates across 17 European countries. J. Child Psychol. Psychiatry Allied Discip..

[12] Fonseca-Pedrero E. (2018). Bienestar Emocional en Adolescentes Riojanos.

[13] Lim K.-S., Wong C., McIntyre R.S., Wang J., Zhang Z., Tran B.X., Tan W., Ho C.S., Ho R. (2019). Global lifetime and 12-month prevalence of suicidal behavior, deliberate self-harm and non-suicidal self-injury in children and adolescents between 1989 and 2018: A meta-analysis. Int. J. Environ. Res. Public Health.

[14] Miranda-Mendizabal A., Castellví P., Parés-Badell O., Alayo I., Almenara J., Alonso I., Blasco M.J., Cebrià A., Gabilondo A., Gili M. (2019). Gender differences in suicidal behavior in adolescents and young adults: Systematic review and meta-analysis of longitudinal studies. Int. J. Public Health.

[15] Fonseca-Pedrero E., Pérez de Albéniz A. (2020). Evaluación de la conducta suicida en adolescentes: A propósito de la escala Paykel de suicidio. Pap. Psicólogo.

[16] Franklin J.C., Ribeiro J.D., Fox K.R., Bentley K.H., Kleiman E.M., Huang X., Musacchio K.M., Jaroszewski A.C., Chang B., Nock M.K. (2017). Risk factors for suicidal thoughts and behaviors: A meta-analysis of 50 years of research. Psychol. Bull..

[17] Ribeiro J.D., Franklin J.C., Fox K.R., Bentley K.H., Kleiman E.M., Chang B.P., Nock M.K. (2016). Self-injurious thoughts and behaviors as risk factors for future suicide ideation, attempts, and death: A meta-analysis of longitudinal studies. Psychol. Med..

[18] Nock M.K., Green J.G., Hwang I., McLaughlin K.A., Sampson N.A., Zaslavsky A.M., Kessler R.C. (2013). Prevalence, Correlates, and Treatment of Lifetime Suicidal Behavior among Adolescents. JAMA Psychiatry.

[19] Hill R.M., Castellanos D., Pettit J.W. (2011). Suicide-related behaviors and anxiety in children and adolescents: A review. Clin. Psychol. Rev..

[20] O’Neil Rodriguez K.A., Kendall P.C. (2014). Suicidal Ideation in Anxiety-Disordered Youth: Identifying Predictors of Risk. J. Clin. Child Adolesc. Psychol..

[21] McCutcheon A.L. (1987). Latent Class Analysis.

[22] Lubke G.H., Muthén B. (2005). Investigating population heterogeneity with factor mixturemodels. Psychol. Methods.

[23] De Luca S., Yan Y., Lytle M., Brownson C. (2014). The associations of race/ethnicity and suicidal ideation among college students: A latent class analysis examining precipitating events and disclosure patterns. Suicide Life Threat. Behav..

[24] Van der Stoep A., McCauley E., Flynn C., Stone A. (2009). Thoughts of death and suicide in early adolescence. Suicide Life Threat. Behav..

[25] Judd F., Jackson H., Komiti A., Bell R., Fraser C. (2012). The profile of suicide: Changing or Changeable?. Soc. Psyquiatry Psychiatr. Epidemiol..

[26] Lo Y., Mendell N.R., Rubin D.B. (2001). Testing the number of components in a normal mixture. Biometrika.

[27] Thompson M., Kuruwita C., Foster E.M. (2009). Transitions in suicide risk in a nationally representative sample of adolescents. J. Adolesc. Health.

[28] Li Y., Aggen S., Shi S., Gao J., Tao M., Zhang K., Wang X., Gao C., Yang L., Liu Y. (2014). Subtypes of major depression: Latent class analysis in depressed Han Chinese women. Psychol. Med..

[29] Pan P., Salum G.A., Gadelha A., Moriyama T., Cogo-Moreira H., Graeff-Martins A.S., Rosário M.C., Polanczyk G.V., Brietzke E., Rohde L.A. (2014). Manic symptoms in youth: Dimensions, latent classes, and associations with parental psychopathology. J. Am. Acad. Child Adolesc. Psychiatry.

[30] Adrian M., Bryant Miller A., McCauley E., Vander Stoep A. (2016). Suicidal ideation in early to middle adolescence: Sex-specific trajectories and predictors. J. Child Psychol. Psychiatry.

[31] Hamza C.A., Willoughby T. (2013). Nonsuicidal self-injury and suicidal behavior: A latent class analysis among young adults. PLoS ONE.

[32] Jiang Y., Perry D.K., Hesser J.E. (2010). Suicide patterns and association with predictors among Rhode Island public high school students: A latent class analysis. Am. J. Public Health.

[33] Xiao Y., Romanelli M., Lindsey M.A. (2019). A latent class analysis of health lifestyles and suicidal behaviors among US adolescents. J. Affect. Disord..

[34] Wong Y.J., Maffini C.S. (2011). Predictors of Asian American Adolescents Suicide Attempts: A Latent Class Regression Analysis. J. Youth Adolesc..

[35] King C.A., Brent D.A., Grupp-Phelan J., Shenoi R., Page K., Matabele-Gittens E.M., Chernick L.S., Melzer-Lange M., Rea M., McGuire T.C. (2019). Five profiles of adolescents at elevated risk for suicide attempts: Differences in mental health service use. J. Am. Acad. Child Adolesc. Psychiatry.

[36] Hayes J.A., Petrovich J., Janis R.A., Yang Y., Castonguay L.G., Locke B.D. (2020). Suicide among college students in psychotherapy: Individual predictors and latent classes. J. Couns. Psychol..

[37] Paykel E.S., Myers J.K., Lindenthal J.J., Tanner J. (1974). Suicidal feelings in the general population: A prevalence study. Br. J. Psychiatry.

[38] Fonseca-Pedrero E., Inchausti F., Pérez-Gutiérrez L., Solana R.A., Ortuño-Sierra J., Lucas-Molina B., Domínguez C., Foncea D., Espinosa V., Gorría A. (2017). Ideación suicida en una muestra representativa de adolescentes españoles. Rev. Psiquiatr. Salud Ment..

[39] Goodman R. (1997). The strengths and difficuties questionnaire: A research note. J. Child Psychol. Psychiatry.

[40] Ortuño-Sierra J., Chocarro E., Fonseca-Pedrero E., Riba S.S.I., Muñiz J. (2015). The assessment of emotional and Behavioural problems: Internal structure of The Strengths and Difficulties Questionnaire. Int. J. Clin. Health Psychol..

[41] Cummins R.A., Lau A.L.D. (2005). Personal Wellbeing Index—School Children.

[42] Ebesutani C., Regan J., Smith A., Reise S., Higa-Mcmillan C., Chorpita B.F. (2012). The 10-Item Positive and Negative Affect Schedule for Children, Child and Parent Shortened Versions: Application of Item Response Theory for More Efficient Assessment. J. Psychopathol. Behav. Assess..

[43] Gur R.C., Richard J., Calkins M.E., Chiavacci R., Hansen J.A., Bilker W.B., Loughead J., Connolly J.J., Qiu H., Mentch F.D. (2012). Age group and sex differences in performance on a computerized neurocognitive battery in children age 8–21. Neuropsychology.

[44] Moore T.M., Reise S.P., Gur R.E., Hakonarson H., Gur R.C. (2015). Psychometric properties of the penn computerized neurocognitive battery. Neuropsychology.

[45] Ortuño-Sierra J., Aritio-Solana R., Fonseca-Pedrero E. (2020). New Evidences about Subjective Well-Being in Adolescence and Its Links with Neurocognitive Performance. Int. J. Environ. Res. Public Health.

[46] Boyce W., Torsheim T., Currie C., Zambon A. (2006). The Family Affluence Scale as a measure of national wealth: Validation of an adolescent self-report measure. Soc. Indic. Res..

[47] Fonseca-Pedrero E., Lemos-Giráldez S., Paino M., Villazón-García U., Muñiz J. (2009). Validation of the Schizotypal Personality Questionnaire Brief form in adolescents. Schizophr. Res..

[48] Akaike H. (1987). Factor analysis and AIC. Psychometrika.

[49] Schwarz G. (1978). Estimating the dimension of a model. Ann. Stat..

[50] Sciove S.L. (1987). Application of model-selection criteria to some problems in multivariate analysis. Psychometrika.

[51] Ramaswamy V., DeSarbo W.S., Reibstein D.J., Robinson W.T. (1993). An empirical pooling approach for estimating marketing mix elasticities with PIMS data. Mark. Sci..

[52] IBM Corp (2013). Released. IBM SPSS Statistics for Windows, Version 22.0.

[53] Muthén L.K., Muthén B.O. (2010). Mplus User’s Guide Seventh Edition.

[54] Logan J., Hall J., Karch D. (2011). Suicide categories by patterns of known risk factors: A latent class analysis. Arch. Gen. Psychiatry.

[55] Jung S., Lee D., Park S., Hong H.J. (2019). Subtypes of suicidal ideation in Korean adolescents: A multilevel latent profile analysis. Aust. N. Z. J. Psychiatry.

[56] Ma J.S., Batterham P.J., Calear A.L., Han J. (2019). Suicide risk across latent class subgroups: A test of the generalizability of the Interpersonal Psychological Theory of Suicide. Suicide Life Threat. Behav..

[57] Randall J.R., Sareen J., Bolton J.M. (2018). Suicide and all-cause mortality in a high-risk cohort: A latent class approach. Gen. Hosp. Psychiatry.

[58] Brown G.K., Ten Have T., Henriques G.R., Xie S.X., Hollander J.E., Beck A.T. (2005). Cognitive therapy for the prevention of suicide attempts: A randomized controlled trial. JAMA.

[59] Klonsky E.D., May A.M., Saffer B.Y. (2016). Suicide, suicide attempts, and suicidal ideation. Annu. Rev. Clin. Psychol..

[60] Wetherall K., Cleare S., Eschle S., Ferguson E., O’Connor D.B., O’Carroll R.E., O’Connor R.C. (2018). From ideation to action: Differentiating between those who think about suicide and those who attempt suicide in a national study of young adults. J. Affect. Disord..

[61] Ayalon L., Litwin H. (2009). What cognitive functions are associated with passive suicidal ideation? Findings from a national sample of community dwelling Israelis. Int. J. Geriatr. Psychiatry J. Psychiatry Late Life Allied Sci..

[62] Del Rosario Flores-Soto M., Cancino-Marentes M.E., Varela F., del Rocío M. (2018). Revisión sistemática sobre conductas autolesivas sin intención suicida en adolescentes. Rev. Cuba. Salud Pública.

[63] Wilkinson P., Goodyer I. (2011). Non-suicidal self-injury. Eur. Child Adolesc. Psychiatry.

[64] Suárez L.F.G., Hurtado I.C.V., Betancurt L.N. (2016). Revisión de la literatura sobre el papel del afrontamiento en las autolesiones no suicidas en adolescentes. Cuad. Hispanoam. Psicol..

[65] Muñiz J., Fonseca-Pedrero E. (2019). Diez pasos para la construcción de un test. [Ten steps for test development]. Psicothema.

[66] Fernández-Artamendi S., Al-Halabí S., Burón P., Rodríguez-Revuelta J., Garrido M., González-Blanco L., García-Álvarez L., García-Portilla P., Sáiz P. (2019). Prevention of recurrent suicidal behavior: Case management and psychoeducation. Psicothema.

